# Molecular mapping of the broad bean wilt virus 2 resistance locus *bwvr* in *Capsicum annuum* using BSR-seq

**DOI:** 10.1007/s00122-024-04603-2

**Published:** 2024-04-08

**Authors:** Jung-Min Kim, Joung-Ho Lee, Se-Ran Park, Jin-Kyoung Kwon, Na-Young Ro, Byoung-Cheorl Kang

**Affiliations:** 1 Interdisciplinary Program in Agricultural Biotechnology, College of Agriculture and Life Sciences, Seoul National University, 1 Gwanak-ro, Gwanak-gu, Seoul, 08826 Republic of Korea; 2 Department of Plant Science and Plant Genomics and Breeding Institute, College of Agriculture and Life Sciences, Seoul National University, 1 Gwanak-ro, Gwanak-gu, Seoul, 08826 Republic of Korea; 3 Rural Development Administration, National Academy of Agricultural Science, Jeonju, Republic of Korea

## Abstract

**Key message:**

**Bulked segregant RNA seq of pools of pepper accessions that are susceptible or resistant to Broad bean wilt virus 2 identifies a gene that might confer resistance to this devastating pathogen.**

**Abstract:**

The single-stranded positive-sense RNA virus Broad bean wilt virus 2 (BBWV2) causes substantial damage to pepper (*Capsicum annuum*) cultivation. Here, we describe mapping the BBWV2 resistance locus *bwvr* using a F_7:8_ recombinant inbred line (RIL) population constructed by crossing the BBWV2-resistant pepper accession ‘SNU-C’ with the susceptible pepper accession ‘ECW30R.’ All F_1_ plants infected with the BBWV2 strain PAP1 were susceptible to the virus, and the RIL population showed a 1:1 ratio of resistance to susceptibility, indicating that this trait is controlled by a single recessive gene. To map *bwvr*, we performed bulked segregant RNA-seq (BSR-seq). We sequenced pools of resistant and susceptible lines from the RILs and aligned the reads to the high-quality ‘Dempsey’ reference genome to identify variants between the pools. This analysis identified 519,887 variants and selected the region from 245.9–250.8 Mb of the Dempsey reference genome as the quantitative trait locus region for *bwvr*. To finely map *bwvr*, we used newly designed high-resolution melting (HRM) and Kompetitive allele specific PCR (KASP) markers based on variants obtained from the BSR-seq reads and the PepperSNP16K array. Comparative analysis identified 11 SNU-C-specific SNPs within the *bwvr* locus. Using markers derived from these variants, we mapped the candidate *bwvr* locus to the region from 246.833–246.949 kb. SNU-C-specific variants clustered near DEM.v1.00035533 within the *bwvr* locus. DEM.v1.00035533 encodes the nitrate transporter NPF1.2 and contains a SNP within its 5′ untranslated region. The *bwvr* locus, which contains four genes including DEM.v1.00035533, could represent a valuable resource for global pepper breeding programs.

**Supplementary Information:**

The online version contains supplementary material available at 10.1007/s00122-024-04603-2.

## Introduction

Broad bean wilt virus 2 (BBWV2) is a single-stranded positive-sense RNA virus with a bipartite genome. BBWV2, which belongs to the *Fabavirus* genus, infects economically important plants, including pepper (*Capsicum annuum*) (Lee et al. 2000), spinach (*Spinacia oleracea*) (Lee et al. 1979), celery (*Apium graveolens*) (Hahm et al. 1998), broad bean (*Vicia faba*) (Stubbs 1947), pea (*Pisium sativum*) (Choi et al. 2001), lily (*Lilium* spp.) (Chang and Chung 1987), and petunia (*Petunia* spp.) (Lesemann 1996). The virus is transmitted in a nonpersistent manner by aphids, specifically *Aphis gossypii* or *Myzus persicae*, causing symptoms such as mosaic patterns, yellow vein clearing, leaf malformation, wilting, stunting, and chlorosis (Kwak et al. 2016).

Serotyping, conducted using a double immunodiffusion test, helped distinguish BBWV1 and BBWV2 (Uyemoto and Provvidenti 1974). This differentiation was confirmed through coat protein (CP) sequence analysis (Kobayashi et al. 2003). The BBWV2 genome comprises two single-stranded positive-sense RNAs, RNA1 and RNA2, which are approximately 5,960 nucleotides and 3,600 nucleotides long, respectively. The 5’ end is linked to viral genome-linked protein (VPg), while the 3’ end is polyadenylated. Both RNA segments carry a single open reading frame (ORF) that is translated into a single polyprotein. This polyprotein is subsequently cleaved by protease (Pro) within its own structure. The RNA1-derived polyprotein includes five mature proteins: protease cofactor (Co-Pro), NTP-binding motif (NTBM), VPg, Pro, and RNA-dependent RNA polymerase (RdRp). The RNA2-derived polyprotein consists of three immature proteins: movement protein (MP), large coat protein (LCP), and small coat protein (SCP) (Ferrer et al. 2011; Goldbach et al. 1995; Kwak et al. 2013b).

Pepper (*Capsicum* spp.) belongs to the Solanaceae family, along with other domesticated crops such as tomato (*Solanum lycopersicum*) and potato (*Solanum tuberosum*). This globally important vegetable crop is commonly cultivated in Korea for use as a spice and flavoring agent. However, viral diseases pose a critical challenge to commercial pepper cultivation, as they are difficult to control, causing reduced yields and requiring vector management (Kim et al. 2014). Several major pepper viruses have been identified in Korea, including BBWV2, cucumber mosaic virus (CMV), pepper mottle virus (PepMoV), pepper mild mottle virus (PMMoV) and potato virus Y (PVY) (Kwak et al. 2013a). Among these, the aphid-transmitted RNA viruses BBWV2 and CMV are responsible for 71.4 and 73.3% of infections in Korean pepper fields, respectively. The co-infection rate reached 55.1% in 2016 (Kwon et al. 2018). A synergic interaction was observed between CMV and BBWV2 in co-infected *Nicotiana benthamiana* plants using a visual tracking method (Kwon et al. 2023). This study demonstrated that BBWV2 facilitates the cell-to-cell movement of CMV in upper younger leaves and that the accumulation of BBWV2 increases in plants co-infected with CMV. These findings highlight the urgent need to identify a source of resistance to BBWV2 in pepper; no genes conferring such resistance have thus far been identified.

Bulked segregant analysis (BSA) is an efficient method for mapping quantitative trait loci (QTLs) or genes by comparing the variants found in bulks with contrasting phenotypes. This method was first used with two bulked DNA samples from individuals with identical traits but arbitrary differences at all unlinked regions (Michelmore et al. 1991). By leveraging polymorphic markers, researchers successfully identified a gene for resistance to downy mildew in lettuce. Bulked segregant RNA sequencing (BSR-seq) analysis is a modified BSA method that uses RNA-seq reads instead of DNA-seq reads (Liu et al. 2012). BSR-seq leverages global patterns of gene expression to identify variants between two different samples. This method has been successfully utilized to identify QTLs and genes in tomato and pepper (Byun et al. 2022; Lin et al. 2023; Yang et al. 2020).

In this study, we screened 1,765 pepper accessions for resistance to BBWV strain PAP1 and identified 30 resistant accessions, including *C. annuum* ‘SNU-C’. Screening of *C. annuum* ‘ECW30R’ and F_1_ (SNU-C × ECW30R) plants revealed that the resistance gene in SNU-C behaves as a recessive gene. In a F_7:8_ population of 148 recombinant inbred lines (RILs) derived from a cross between SNU-C and ECW, the resistant-to-susceptible ratio of the RILs was 1:1, indicating that the trait is governed by a single gene. To locate the resistance locus *bwvr*, we conducted BSR-seq analysis and determined that *bwvr* is located between 245 and 251 Mb of the ‘Dempsey’ reference genome. Using markers designed from SNU-C-specific variants, we successfully narrowed the *bwvr* locus to a 115-kb region and identified a candidate gene for BBWV PAP1 resistance.

## Materials and methods

### Plant materials

A total of 1,765 genetic resource accessions (GRAs), including 1,105 *C. annuum*, 306 *C. chinense*, 220 *C. frutescens*, 65 *C. baccatum*, 3 *C. chacoense*, and 66 unknown *Capsicum* species, were obtained from the National Institute of Agricultural Science, Rural Development Administration, Republic of Korea (RDA). The *C. annuum* cv. SNU-C (‘SNU-C’) was used as a resistant control for broad bean wilt virus 2 (BBWV2), while the *C. annuum* cv. Early Cal Wonder 30R (‘ECW30R’) was used as the susceptible control. The recombinant inbred lines (RILs) resulting from a cross between SNU-C and ECW30R, referred to as the SERIL mapping population, were used for marker validation. A total of 148 F_7:8_ SERILs were screened to map the BBWV2 resistance gene.

### Virus inoculation

Full-length infectious cDNA clones of BBWV2 strain PAP1, which causes severe symptoms, were provided by Professor Jang-Kyun Seo (Seoul National University). An *Agrobacterium* strain containing the infectious clone was cultivated in LB broth and stored in a –70 ℃ freezer.

BBWV PAP1 was maintained in *Nicotiana benthamiana*. Three-week-old *N. benthamiana* plants were inoculated with the *Agrobacterium* strain via infiltration using a needless syringe. Leaves were collected from the plants at 21 days post-inoculation (dpi) and ground in 0.1 M potassium phosphate buffer (1 g sample per 10 ml buffer). The resulting material was used to inoculate the cotyledons of 2 week-old pepper plants by rubbing the leaves with carborundum powder.

### Screening of viral symptoms

The viral symptoms of BBWV2 PAP1 were assessed 2 weeks after inoculation. Observations were made on the upper leaves to identify symptomatic indicators, and the severity of the symptoms was quantified by measuring the sizes of the lesions, scaled as follows: 0 (no symptoms), 1 (lesions covering 0–10% of the leaf), 2 (10–30% of the leaf), 3 (30–50% of the leaf), and 4 (> 50% of the leaf). Ten plants of each GRA and SERIL were scored, and lines with mean scores < 1 were considered to be resistant. These putatively resistant lines were validated by double antibody sandwich enzyme-linked immunosorbent assay (DAS ELISA), along with the lines with high standard deviations. ELISA was performed using an ELISA Reagent Set for Broad bean wilt virus 1, 2 (Agdia, Elkhart, IN, USA). SNU-C and ECW30R were used as the negative and positive controls, respectively.

### RNA isolation and sequencing

From the SERILs, 14 resistant lines and 14 susceptible lines exhibiting the lowest standard deviations were selected for RNA sequencing (RNA-seq). Leaf samples were collected from the upper leaves of the SERILs at 14 dpi with BBWV2. Total RNA was extracted from the samples using a TaKaRa MiniBEST Plant RNA Extraction Kit (Takara, Kusatsu, Japan) according to the manufacturer’s instructions. After a quality check, the RNA samples were divided into three resistant pools (*R* pools) and three susceptible pools (*S* pools). The extracted total RNA pools were sent to Macrogen Inc. (Seoul, Korea) for sequencing. A TruSeq RNA sample preparation kit (Illumina, USA) was used to construct a cDNA library, and paired-end reads were generated using the Illumina NovaSeq6000 platform (Illumina, USA).

### Sequence alignment and variant calling

The RNA-seq reads were trimmed with Fastp v0.12.4 (Chen et al. 2018). The trimmed reads were aligned to the ‘Dempsey’ reference genome (Lee et al. 2022) using hisat2 v.2.2.1 (Kim et al. 2019). Variants for each RNA-seq pool were identified using gatk v4.1.7.0 (Van der Auwera and O’Connor 2020). The alignment files were split using the gatk SplitNCigarReads tool, and variant calling was performed using the gatk HaplotypeCaller tool. Single-nucleotide polymorphisms (SNPs) were selected from among the variants using the gatk SelectVariants tool and filtered with the gatk VariantFiltration tool, applying the following options: QD < 2.0, QUAL < 30.0, SOR > 3.0, FS > 60.0, MQ < 40.0, MQRankSum < –12.5, ReadPosRankSum < –8.0. Insertion/deletion variants (indels) were filtered with different options: QD < 2.0, QUAL < 30.0, FS > 200.0, ReadPosRankSum < –20.0. Only biallelic SNPs were used for further analysis.

### BSR-seq analysis

The acquired variants were subjected to BSR-seq analysis using the QTLseqr R package (Mansfeld and Grumet 2018). SNPs and indels were filtered based on the following criteria: a reference allele frequency within the range of 0.3 to 0.7, a minimum total depth of 100 reads, a maximum total depth of 5,000 reads, and a genotype quality threshold of at least 99. Filtered variants were then employed to calculate G’ value and tricube-smoothed ΔSNP-index with a window size of 4 Mb. SNPs and indels with a *G*’ value exceeding the threshold of a *q* value of 0.01, or with a ΔSNP-index surpassing the 99% confidence interval, were considered to be significant QTLs.

### Variant annotation and marker development for fine mapping

SNPs and indels within the QTL region were annotated with SnpEff (Cingolani et al. 2012). Annotated SNPs from the PepperSNP16K array (Hulse-Kemp et al. 2016) within the QTL region were also used for subsequent analysis. High-resolution melting analysis (HRM) markers were designed, considering the annotation and the distance between the variants. These markers were tested on an F_7:8_ SERIL population (*n* = 90) using a Rotor-Gene 6000 real-time PCR machine (Qiagen, Hilden, Germany), and the results were analyzed using Rotor-Gene *Q* series software version 2.1.0.

To narrow the mapping region, variants within range of the two nearest markers were compared across seven pepper genomes including *C. annuum* ‘Dempsey,’ ‘Micropep,’ ‘CV3’, ‘Maor,’ ‘Perennial,’ ‘Thaihot,’ and ‘ECW’ (Lee et al. 2022). Among these, eight variants were chosen for Kompetitive allele-specific PCR (KASP) analysis. KASP markers were tested on an F_7:8_ SERIL population (*n* = 148) using the quantstudio 3 real-time PCR System (ThermoFisher, Waltham, MA, USA), and the results were analyzed using quantstudio design & analysis software.

### Analysis of differentially expressed genes

Analysis of differentially expressed genes (DEGs) was conducted using the DESeq2 *R* package (Love et al. 2014). Transcript count data from three replicates each of both the *R* and *S* pools were used as input. DEGs meeting the criteria of an adjusted *p* value < 0.05 and a fold change ≥ 2 were retained. These filtered DEGs were functionally annotated using gene ontology terms (GO). The annotated DEGs were further enriched by single enrichment analysis using the web-based tool AgriGO v2 (Tian et al. 2017). The filtered DEGs were also subjected to pathway annotation using ShinyGO v0.77 (Ge et al. 2020).

## Results

### BBWV2 PAP1 screening in parental lines and the mapping population

**Fig. 1:**
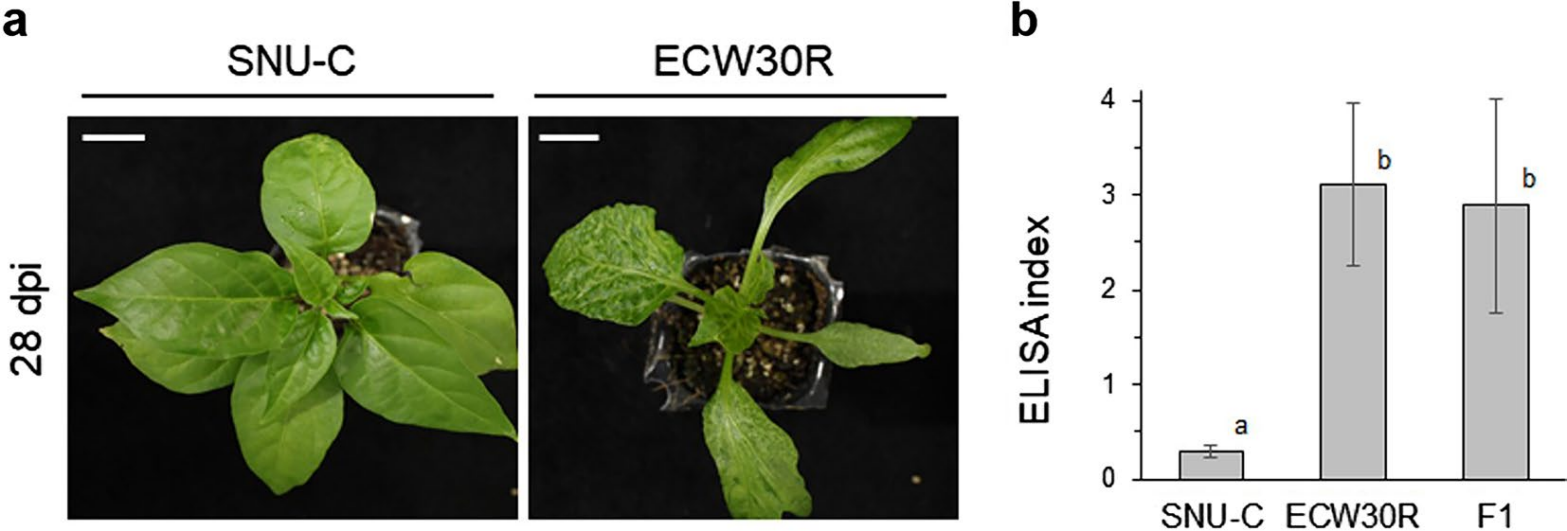
Comparison of the phenotypes of SNU-C and ECW30R infiltrated with BBWV2 PAP1. **a** Disease symptom of SNU-C and ECW30R. ECW30R exhibits mosaic patterning, yellow vein clearing, and leaf malformation, while SNU-C remains asymptomatic at 28 days post-inoculation (dpi) with BBWV2 PAP1; scale bar = 1 cm; **b** ELISA results of BBWV2 PAP1 infiltrated accessions (*n* = 9 per accession). ELISA index indicates that both ECW30R and F_1_ plants are susceptible to BBWV2 PAP1. Different letters indicate significant differences between SNU-C, ECW30R, and F_1_ according to Duncan’s multiple range test (*p* ≤ 0.05) following One-way ANOVA analysis; error bars indicate standard deviation (SD)

The resistance of the 1,765 GRAs provided by the RDA to BBWV2 PAP1 was tested (Supplementary Table 1). Of these, 30 accessions were identified as resistant, including 27 *C. annuum* accessions, 2 unknown *Capsicum* species, and 1 *C. frutescens* accession. These accessions were confirmed to be resistant using ELISA (Supplementary Fig. 1). These accessions included *C. annuum* SNU-C, and therefore, we were able to utilize the RIL population derived from a cross between SNU-C and ECW30R (F_7:8_ SERIL). To evaluate the suitability of the RIL population for analysis, the disease responses of SNU-C, ECW30R, and F_1_ plants derived from a cross between SNU-C and ECW30R to BBWV2 PAP1 were examined. ECW30R displayed symptoms including mosaic patterns, yellow vein clearing, and leaf malformation, while SNU-C remained asymptomatic (Fig. 1a). Virus accumulation was not observed in SNU-C, as confirmed by ELISA (Fig. 1b). F_1_ plants were susceptible to BBWV2 PAP1, suggesting that the allele for the BBWV2 PAP1 resistance gene is likely recessive.

**Fig. 2:**
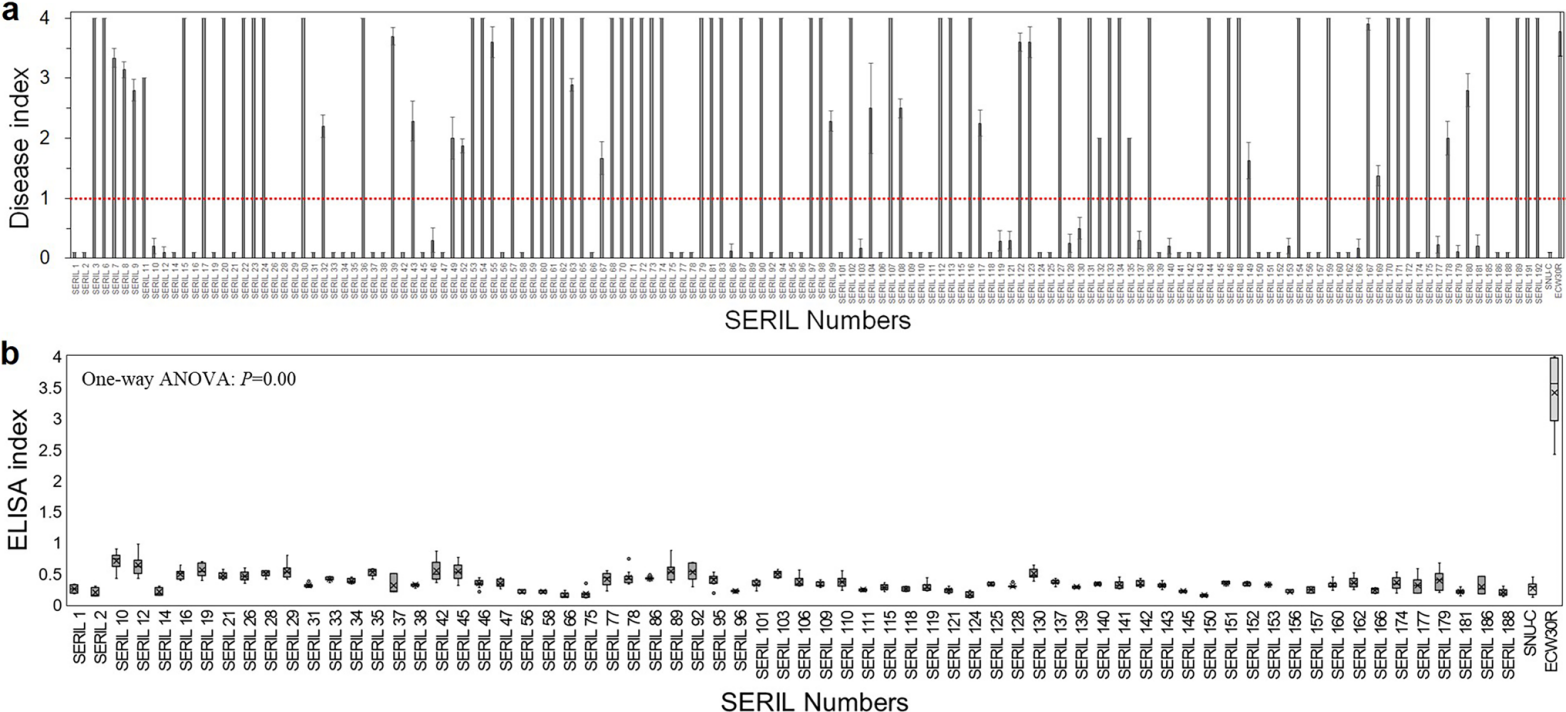
Disease index and ELISA results of SERILs infiltrated with BBWV2 PAP1. **a** BBWV2 disease index in 148 SERILs. Disease index was determined based on the size of the lesion (0: no symptoms, 1: lesions covering 0–10% of the leaf, 2: 10–30% of the leaf, 3: 30–50% of the leaf, 4: > 50% of the leaf). The red dotted line indicates the threshold used to evaluate whether the line is resistant or susceptible to BBWV2 PAP1. SERILs with an average disease index < 1 were considered to be resistant. Bars display the mean disease index for each RIL; error bars represent SE. **b** Box plot of ELISA results from 68 putatively resistant lines. All lines that were resistant according to disease index scoring were confirmed to be resistant by ELISA, with SNU-C and ECW30R serving as controls. The box and lines indicate the median, first quartile, and third quartile values, while the x mark shows the mean value. One-way ANOVA analysis followed by post hoc Tukey–Kramer’s test was applied for statistical analysis, comparing every accession to ECW30R. All comparisons resulted in an adjusted *p* value < 0.001

To further characterize the resistance allele, the SERIL mapping population (*n* = 148) were inoculated with BBWV2 PAP1 (Fig. 2a; Supplementary Table 2). Sixty-eight resistant lines were identified and validated by ELISA (Fig. 2b). A chi-squared test indicated that the segregation of the resistant and susceptible phenotypes followed a 1:1 ratio (Table 1). These results suggest that resistance to BBWV2 PAP1 is controlled by a single gene; the resistance locus will be referred to as *bwvr* hereafter.

**Table 1:** Phenotypic segregation analysis of SNU-C, ECW30R, F_1_ plants, and RILs inoculated with BBWV2 PAP1

Population	Number of Plants			*χ*2 (1:1)*
	Total	Resistant	Susceptible	
SNU-C	9	9	0	
ECW30R	9	0	9	
F_1_ (SNU-C × ECW30R)	9	0	9	
F_7:8_ SERILs	148	68	80	*p* = 0.3239

^*^p value calculated from chi-squared tests

### RNA-seq and BSA

**Fig. 3:**
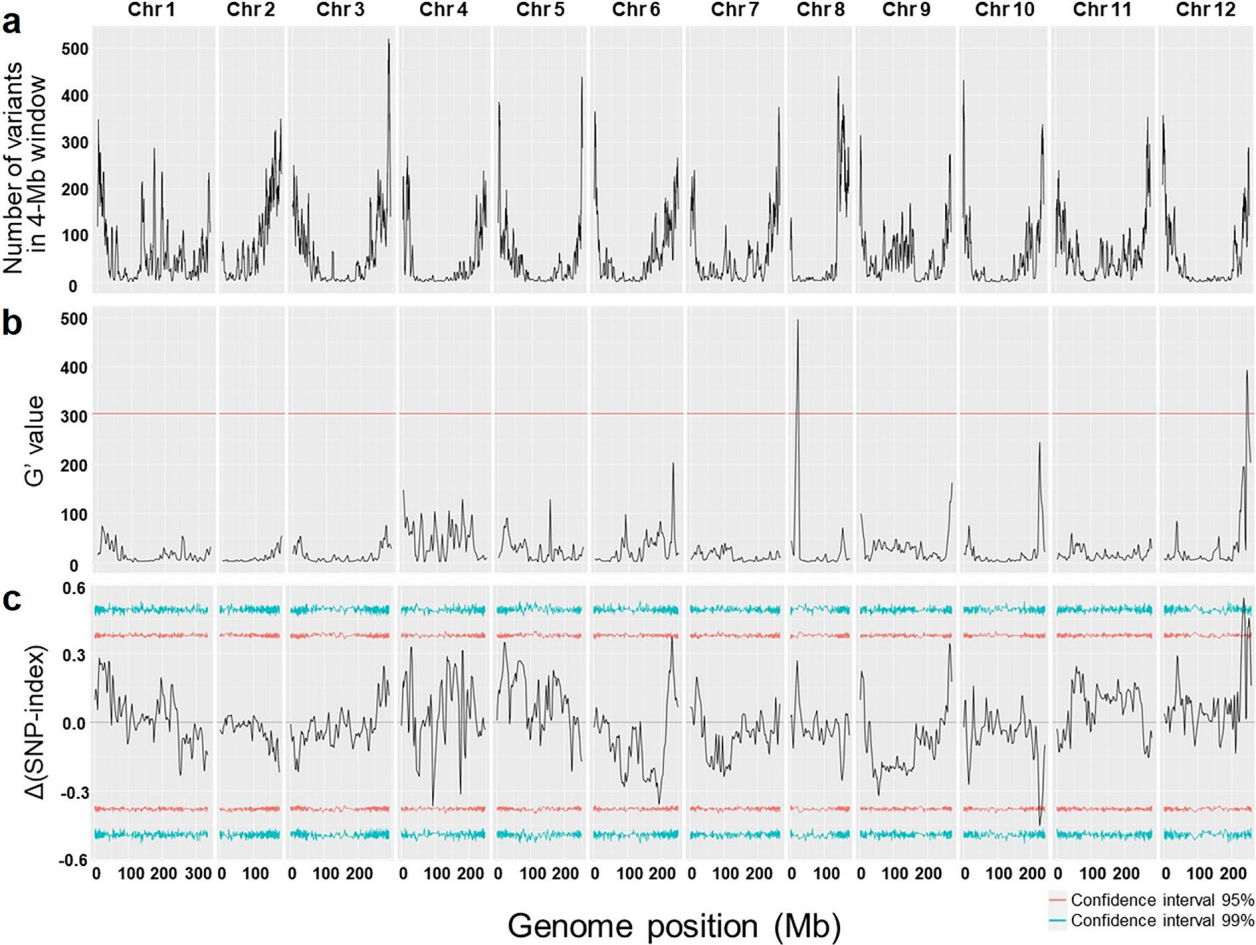
BSR-seq analysis of bulked segregant RNA-seq reads. **a** Plot showing the number of variants (SNPs and indels) in a window size of 4 Mb. On average, each chromosome contained 2,495 variants, with chr3 having the highest count (3,474) and chr4 having the lowest count (1,890). **b** Plot showing the peak *G*’ values. The red line indicates the significant G’ value threshold (*q* value = 0.01). Two peaks were identified as significant, located on chr8 (18.4–19.6 Mb) and chr12 (245.8–250.8 Mb). **c** Plot showing the peaks of the tricube-smoothed ΔSNP-index. The red and blue lines indicate a confidence interval threshold of 95% and 99%, respectively. Two peaks passed the 95% threshold, located on chr6 (231.2–232.1 Mb) and chr12 (231.2–254.2 Mb). Additionally, one peak passed the 99% threshold, located on chr12 (235.8–237.9 Mb)

Of the 148 SERILs examined, 14 susceptible and 14 resistant lines were selected for RNA-seq. These lines were bulked into three susceptible pools (*S* pools) and three resistant pools (*R* pools). On average, 6.8 Gb data were obtained, resulting in 68,154,477 reads from the sequencing of these pools (Supplementary Table 3). These reads were aligned to the high-quality reference genome Dempsey. Subsequently, 419,831 SNPs and 166,205 indels were obtained by variant calling. These variants were filtered using different criteria, and only biallelic variants were retained, resulting in 368,335 SNPs and 151,552 indels. After merging and further filtering based on read depth, reference allele frequency, and genotype quality, a final set of 29,937 variants for BSR-seq analysis (chr1: 3,206 variants; chr2: 2,765; chr3: 3,474; chr4: 1,890; chr5: 2,112; chr6: 2,342; chr7: 2,559; chr8: 2,466; chr9: 2,566; chr10: 2,013; chr11: 26,00; chr12: 1,944) were obtained (Fig. 3a). By calculating the *G*’ value and tricube-smoothed ΔSNP-index in a 4-Mb window, two significant peaks of *G*’ value that exceeded the *G*’ value threshold of 300 were identified (Fig. 3b) and three significant peaks of tricube-smoothed ΔSNP-index that surpassed the 95 and 99% confidence intervals (Fig. 3c). To select the most significant QTL, peaks from two different analyses were compared, each using G’ value and ΔSNP-index. This comparison revealed a peak on chromosome 12 that overlapped between the two analyses located at position 245.9 to 250.8 Mb in the Dempsey reference genome. This region is referred to as the bwvr-qtl region hereafter.

### Fine mapping of the *bwvr* locus

**Fig. 4:**
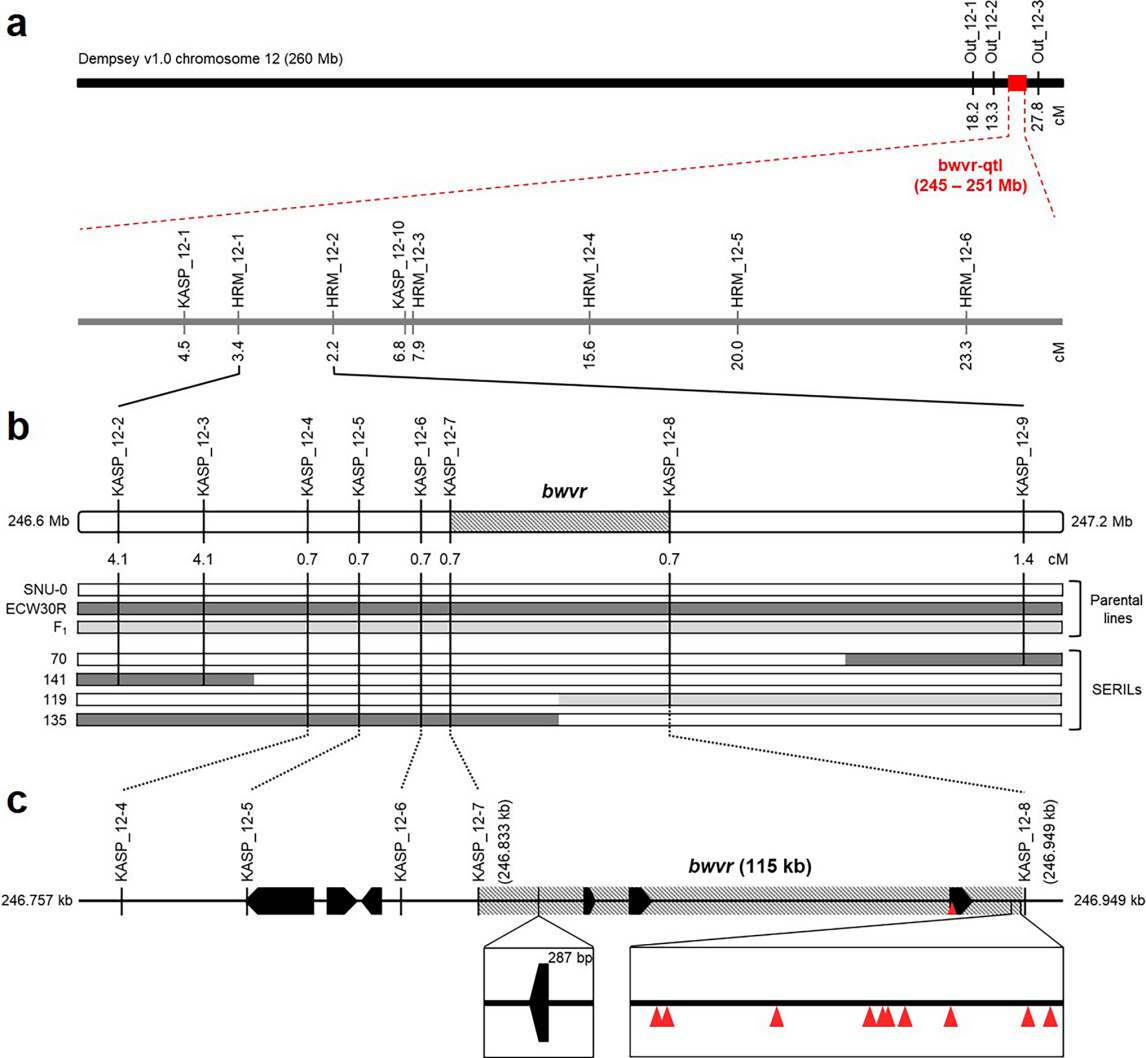
Fine mapping of the *bwvr* locus. **a** The QTL of *bwvr* identified by BSR-seq. Markers outside the significant *G*’ peak region (245.8–250.8 Mb) and within the significant ΔSNP-index peak region (231.2–254.2 Mb) were tested. The recombination rate indicates that the *bwvr* locus lies within the QTL. Six HRM markers and one KASP marker were developed for rough mapping within the QTL region using 90 SERILs. **b** Fine mapping of the *bwvr* locus between HRM_12-1 (3.4 cM) and HRM_12-2 (2.2 cM). These two flanking markers were developed into KASP markers. Eight KASP markers were tested on 148 SERILs. Four SERILs with recombinants in the fine mapping region are shown together with the parental lines and F_1_. Box with diagonal lines between KASP_12-7 and KASP_12-8 indicates the final candidate region of the *bwvr* locus (115 kb). **c** Genes located in the delimited region. Seven annotated genes were located in the above region of markers, with one recombinant, and four of these genes were located in the candidate region of the *bwvr* locus (highlighted with diagonal lines). The black box with arrows indicates the annotated genes in the Dempsey reference genome. The red arrows indicate the SNU-C-specific variants in the *bwvr* locus (color figure online)

To determine the location of the BBWV2 resistance gene locus, SNP and indel markers were designed in the vicinity of the bwvr-qtl region (Supplementary Table 4, 5). Initially, three HRM markers (Out_12-1, Out_12-2, and Out_12-3) located outside the bwvr-qtl but within the ΔSNP-index peak region (95% interval) were tested on 90 SERILs. A decrease in recombination rate was observed as the markers approached the bwvr-qtl region, indicating that the *bwvr* locus is indeed located within the bwvr-qtl region. Six HRM markers and two KASP markers were tested within this region on 90 SERILs (Fig. 4a). HRM12-1 and HRM12-2 showed the fewest recombinants (three and two recombinants, respectively). This region, located at position 246.6 to 247.2 Mb in the Dempsey reference genome, was specifically targeted for fine mapping of the *bwvr* locus using eight KASP markers. These KASP markers were designed using SNPs from the PepperSNP16K array in conjunction with the SNPs and indels discovered by BSR-seq. When designing the markers based on BSR-seq-derived variants, we aligned these variants against seven long-read sequenced genomes: *C. annuum* Dempsey, ‘Micropep,’ ‘CV3,’ ‘Maor,’ ‘Perennial,’ ‘ThaiHot’ (Lee et al. 2022), and ECW (unpublished data). All seven of these pepper accessions are susceptible to BBWV PAP1. Therefore, alleles present in SNU-C and differ in the seven other pepper genomes were selected for marker analysis (Supplementary Table 6).

Among the eight KASP markers tested, five exhibited recombination in a single plant among the 148 RILs (Fig. 4b). Specifically, KASP_12-4, KASP_12-5, KASP_12-6, and KASP_12-7 exhibited recombination in SERIL 135, while KASP 12–8 exhibited recombination in SERIL 119. These results indicate that the recombination region is located between these two markers. Consequently, it is likely that the *bwvr* locus is located between the recombination regions of two markers, KASP_12-7 and KASP_12-8, spanning from 246.833 to 246.949 kb in the Dempsey reference genome.

### Candidate genes in the *bwvr* locus

Candidate genes within the *bwvr* locus were identified based on the annotation of the Dempsey reference genome (Fig. 4c). Seven genes (DEM.v1.00035525, DEM.v1.00035526, DEM.v1.00035527, DEM.v1.00035530, DEM.v1.00035531, DEM.v1.00035532, and DEM.v1.00035533) were located within the single recombinant region of five markers (Table 2). Among these markers, KASP12-5 was located within a gene, while the four other markers were located in intergenic regions. Five of the annotations were from *Arabidopsis thaliana*, one from *Solanum tuberosum*, and one from *Escherichia coli*. Four of the seven genes (DEM.v1.00035530, DEM.v1.00035531, DEM.v1.00035532, and DEM.v1.00035533) were located within the *bwvr* locus. Three of the genes were annotated as encoding the same type of protein, NRT1/PTR FAMILY 1.2 proteins, albeit with different identities. Additionally, one of the genes was predicted to be the transposon RE1, which was the only gene that showed significant differences in expression, as calculated by normalized fragments per kilobase of transcript per million (FPKM) value.

**Table 2:** Annotated genes in the mapping region and their fragments per kilobase of transcript per million (FPKM) values

Gene Name	Species	Annotation	Protein size	FPKM	
				*R*_Pool	*S*_Pool
*DEM.v1.00035525*	*Solanum tuberosum*	Similar to PFP-ALPHA: Pyrophosphate–fructose 6-phosphate 1-phosphotransferase subunit alpha	578	12.37	13.20
*DEM.v1.00035526*	*Escherichia coli* (strain K12)	Similar to yjbQ: UPF0047 protein YjbQ	182	97.86	97.69
*DEM.v1.00035527*	*Arabidopsis thaliana*	Similar to At1g76660: Uncharacterized protein At1g76660	445	22.59	18.20
***DEM.v1.00035530***	***Arabidopsis thaliana***	**Similar to RE1: Retrovirus-related Pol polyprotein from transposon RE1**	**95**	**0.00**	**0.33**
***DEM.v1.00035531***	***Arabidopsis thaliana***	**Similar to NPF1.2: Protein NRT1/PTR FAMILY 1.2**	**536**	**3.26**	**4.56**
***DEM.v1.00035532***	***Arabidopsis thaliana***	**Similar to NPF1.2: Protein NRT1/PTR FAMILY 1.2**	**474**	**7.66**	**6.02**
***DEM.v1.00035533***	***Arabidopsis thaliana***	**Similar to NPF1.2: Protein NRT1/PTR FAMILY 1.2**	**602**	**57.86**	**61.95**

Genes in the *bwvr* locus are indicated in bold

One hundred and forty-one variants were identified at the *bwvr* locus. Among these, 130 variants did not show correlations with the phenotypes of eight accessions (SNU-C, Dempsey, Micropep, CV3, Maor, Perennial, ThaiHot and ECW). Eleven SNU-C-specific variants that did show correlations with the phenotypes of these eight accessions were annotated using SnpEff. Ten of the variants were localized to intergenic regions, and one was identified as an intragenic variant within the gene DEM.v1.00035533 (Fig. 4c). This variant was located in the 5′ untranslated region (UTR) of this gene, with a sequence substitution from G to T. Differences in the number of transcripts were identified at the point of variation (Supplementary Fig. 2). Variants in the 5′ UTR were manually identified via the alignment of SNU-C and ECW sequences (Supplementary Fig. 3). Considering that the SNU-C-specific SNPs are clustered around DEM.v1.00035533 and that the transcription pattern changes in the region containing the SNP, it is likely that DEM.v1.00035533 is *bwvr*.

### DEG analysis and annotation

**Fig. 5:**
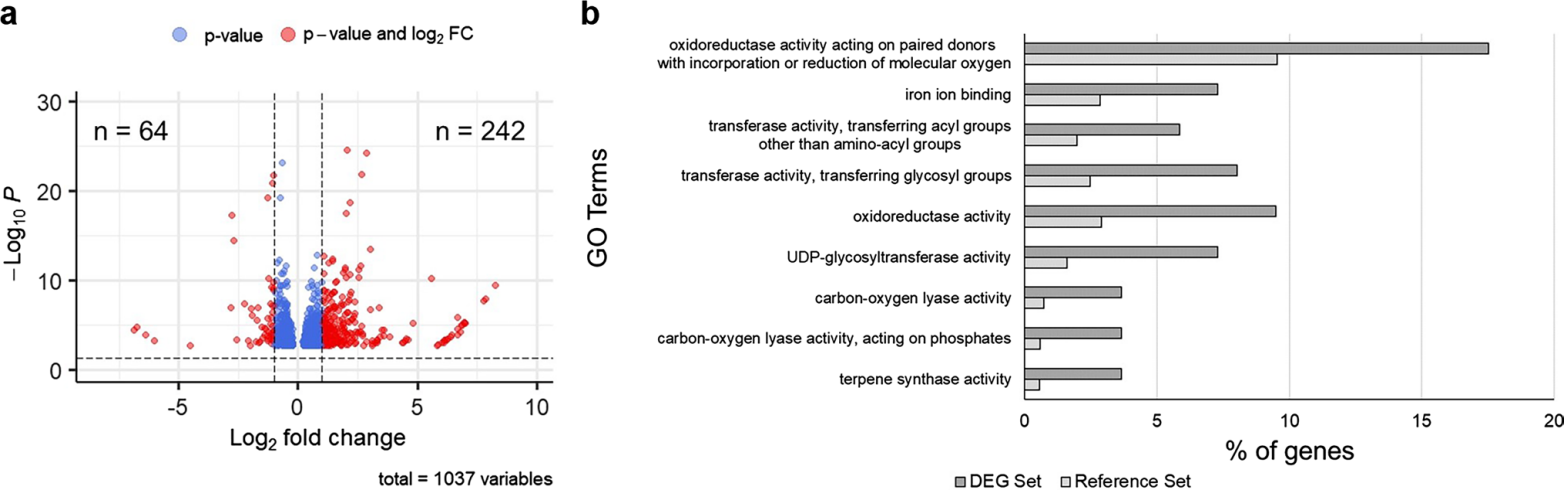
DEG analysis of transcripts identified by BSR-seq. **a** Volcano plot of DEGs obtained from the DESeq2 *R* package. 242 genes were upregulated and 64 genes were downregulated in the *S* pool. The horizontal dotted line is the threshold for *p* value of 0.05, and the vertical dotted line is the threshold for a log_2_ fold change greater than 1 and less than − 1. Red dots indicate DEGs that passed both thresholds, while blue dots indicate DEGs that passed only the *p* value threshold. **b** Graphical summary of GO enrichment analysis of the upregulated DEGs in the *S* pool. The white bar represents the percentage of DEGs with a specific molecular function among upregulated DEGs, while the gray bar represents the percentage of genes with a specific molecular function among the total genes annotated in the reference genome. The enriched GO terms were ordered by the fold changes in the DEG set compared to the reference set (color figure online)

The BSR-seq reads, representing pooled transcripts, were subjected to DEG analysis. The analysis resulted in 306 DEGs that met the threshold of *p* value below 0.05 and log_2_ fold change difference exceeding 1 or falling below − 1. Among these DEGs, 242 were upregulated and 64 were downregulated in the *S* pools (Fig. 5a). The upregulated DEGs in the *S* pools were subjected to GO enrichment analysis for functional annotation. Nine molecular function GO terms were significantly enriched among these DEGs (Fig. 5b). Oxidoreductase activity and transferase activity were among the enriched terms and are associated with defense responses in plants, suggesting that the susceptible SERILs recognize pathogen invasion. The results of enrichment analysis of functional pathways using ShinyGO support the hypothesis that susceptible plants recognize pathogen invasion, which induces plant defense responses (Supplementary Fig. 4). These findings provide insights into the function of the recessive resistance allele *bwvr* as a player in the multilayered plant immunity response.

## Discussion

BBWV2 poses a significant threat to various economically important crops due to its broad host range. BBWV2 ranks among the major viruses in pepper in Korea along with CMV, PepMoV, PMMoV, and PVY. When co-infected with CMV, BBWV2 exhibits a synergic interaction that increases viral accumulation, accelerates cell-to-cell movement, and increases symptom severity. The need for a source of resistance to BBWV2 is increasing, but to date, no genes have been identified that confer resistance to BBWV2 in pepper.

In this study, a total of 1,765 GRAs were screened, and 30 pepper accessions were identified as resistant to BBWV2, a number higher than expected. Of these, 27 were *C. annuum*, and we are currently working on the comparison of these *C. annuum* originated resistance with *bwvr*. The resistance sources originating from *C. chinense* and *C. frutescens* are also interesting, and we will analyze these after sufficiently understanding the resistance sources of *C. annuum*.

Using BSR-seq, the BBWV2 resistance locus *bwvr* was successfully mapped. BSR-seq is an efficient tool for identifying significant variants that correlate with different phenotypes. By comparing the bulk of RNA pooled from each phenotype, variants present in transcripts are selectively revealed. In this manner, there is a greater chance that the variants are actually related to a transcriptional change compared to DNA-based analysis. Genotyping-by-sequencing (GBS) was previously used in an attempt to identify this locus prior to BSR-seq, but the SNP variation revealed by GBS was insufficient to identify the *bwvr* locus on chromosome 12. We believe that the high frequency of recombination in the terminal region of chromosome 12, which can be confirmed from comparing the physical distances and recombination frequencies of markers used in this study, is responsible for this failure. BSR-seq analysis identifies the variants in transcripts, which are more tightly linked to the gene of interest than are variants randomly identified by GBS. Therefore, we were able to identify the *bwvr* locus using BSR-seq analysis.

Comparative analysis of *C. annuum* genomes enabled us to identify SNU-C-specific variants from among a large number of variants. Variants were filtered from BSR-seq reads and the PepperSNP16K array using the genotypes of seven *C. annuum* accessions that were susceptible to BBWV2 PAP1. This analysis not only filtered out the variants that did not correlate with the phenotypes of eight *C. annuum* accessions (including SNU-C) but also selected variants that are unique to the SNU-C gene pool. Genetically powerful variants that are not present in the susceptible *C. annuum* accessions were utilized for further analysis. Based on these variants, eight KASP markers were designed that efficiently narrowed the location of the *bwvr* locus. Additionally, 11 SNU-C-specific SNPs were selected among 141 BSR-seq-derived variants within the *bwvr* locus using comparative analysis. This information helped us narrow the candidate gene without the need to check all 141 variants.

DEM.v1.00035533 is annotated as “Similar to NPF1.2: Protein NRT1/ PTR FAMILY 1.2,” sharing 46.7% identity with the *Arabidopsis thaliana* NPF1.2 protein. *Arabidopsis* NPF1.2 is a transmembrane transporter that functions as a low-affinity nitrate transporter involved in the xylem-to-phloem transfer of nitrate, facilitating its redistribution in developing leaves (Hsu and Tsay 2013). Additionally, NPF1 family members can serve as dipeptide transporters, facilitating the transport of a broad spectrum of di/tripeptides (Tsay et al. 2007). Phylogenetic analysis of subfamily 1 NPF (NPF1) showed a much greater diversity in *C. annuum* (13 proteins) than in Arabidopsis (3 proteins) (Shoji and Saito 2023). There is a broad spectrum of NRT1/PTR FAMILY substrates, including several phytohormones (the auxins IAA and IBA, abscisic acid, gibberellic acid, and the active jasmonate Ja-Ile) (Corratge-Faillie and Lacombe 2017; Shimizu et al. 2021; Watanabe et al. 2020; Wulff et al. 2019), dicarboxylates (Jeong et al. 2004), chloride (Corratge-Faillie and Lacombe 2017) and potassium (Li et al. 2017), all of which are associated with plant responses to biotic/abiotic stress (Kanstrup and Nour-Eldin 2022). Plant specialized metabolites such as steroidal glycoalkaloids (SGA), a toxic metabolite associated with plant resistance to pathogens in *Solanum* species (Lachman et al. 2001), are transported by the NPF1 protein GORKY (Kazachkova et al. 2021). In addition, the grapevine gene *NFP3.2* is upregulated only when susceptible grapevine is inoculated with the powdery mildew (PM) vector insect. Furthermore, the promoter of this gene induces the expression of a GUS reporter in PM-infected leaves (Pike et al. 2014). The expression of *Arabidopsis Nrt2.6*, which is induced only after inoculation with the phytopathogenic bacterium *Erwinia amylovora*, correlates with the accumulation of reactive oxygen species (ROS) in response to pathogen infection (Dechorgnat et al. 2012). Expression analysis of PVY-inoculated PVY-resistant tobacco (*Nicotiana tabacum*) revealed upregulation of the NRT1/PTR gene *JZ897693* (Chen et al. 2017). Studies on NPF1 provide evidence for a link between NPF1 proteins and plant biotic/abiotic stress responses.

Transporters are often associated with resistance to viruses. One example is HIGH-AFFINITY K + TRANSPORTER 5 (OsHAK5), which regulates rice grassy stunt virus (RGSV) infection in rice (*Oryza sativa*). Disruption of *OsHAK5* facilitates virus accumulation, whereas its overexpression enhances resistance to RGSV infection by a ROS-mediated defense response (Jing et al. 2022). *OsHAK5* expression is induced by the RGSV-encoded P3 protein, indicating that the P3 protein serves as an elicitor for *OsHAK5*. Similarly, the hexose transporter gene *LeHT1* (*SlHT1*), which encodes a hexose transporter and is preferentially expressed in tomato yellow leaf curl virus (TYLCV)-resistant tomato plants, is silenced in resistant tomato plants following TYLCV inoculation (Eybishtz et al. 2010). *LeHT*1-silenced plants display higher viral accumulation and necrosis in response to increased ROS levels. This response is not observed in non-silenced resistant tomato, revealing that *LeHT1* expression is one of the multiple layers of the plant immune response. These findings provide insight into how transporters interact with infecting viruses and confer resistance.

The P3a protein of Brassica yellows virus (BrYV) interacts with membrane proteins in *Arabidopsis* (Liu et al. 2023). P3a is required for the systemic movement of BrYV. A split-ubiquitin-based membrane yeast two-hybrid (MYTH) assay using P3a as bait demonstrated the interaction of P3a with three *Arabidopsis* proteins, AtPUP14, AtGTR1, and AtNRT1.7. AtPUP14 is a purine permease protein, while AtGTR1 and AtNRT1.7 belong to the nitrate/peptide transporter family. P3a hijacks AtGTR1 and AtNRT1.7 from the plasma membrane, leading to their movement to the cytosol. Plant protein hijacking by viruses is generally associated with the plant’s secretory pathway for their efficient replication and spreading (Patarroyo et al. 2012). Thus, a link may exist between viral movement and proteins of the nitrate/peptide transporter family.

Based on findings about NPF1 transporter proteins, we propose a model for the possible mode of action of DEM.v1.00035533 in conferring BBWV2 resistance. We suggest that DEM.v1.00035533 functions in a layer of the plant defense response against BBWV2, similar to *LeHT1*. Although other layers of immunity are activated against BBWV2, such as the ROS response and the jasmonic acid-regulated plant defense response, the defense response cannot effectively halt BBWV2 accumulation if DEM.v1.00035533 is disrupted. This notion is in agreement with the results of DEG analysis, where upregulated DEGs in the *S* pool were related to plant defense responses, such as jasmonic acid induction and response to oxygen-related compounds, yet the virus still accumulated. Alternatively, perhaps DEM.v1.00035533 is involved in the secretory pathway or facilitates the movement of BBWV2. BBWV2 might hijack DEM.v1.00035533 for proliferation, cell-to-cell movement, or systemic movement in the infected plant.

The 5′ UTR plays vital roles in regulating gene transcription and translation. One component involved in translational control is the upstream ORF (uORF). The start codon of the uORF in the 5′ UTR inhibits translation in the downstream ORF. Moreover, the uORF stop codon may act as a premature termination codon, leading to the degradation of mRNA via the nonsense-mediated decay pathway (Hellens et al. 2016). Alternative splicing in the UTR may control translation efficiency by generating mRNA variants with different types of uORFs or riboswitches (Roy and Von Arnim 2013). Various other mechanisms, such as the formation of a ribonucleoprotein complex, pseudo-knot, hairpin structure, or RNA binding protein binding site may also affect mRNA translation in the 5′ UTR (Leppek et al. 2018). The 5′ UTR of DEM.v1.00035533 exhibited approximately 15% sequence variations between SNU-C and ECW. The impact of the variation in the 5′ UTR of DEM.v1.00035533 is yet to be determined; further research is required.

In conclusion, we have mapped the BBWV2 resistance gene *bwvr* to chromosome 12 of pepper using an SERIL population and the BSR-seq method with 0.7 cM flanking markers. The *bwvr* region contains four genes, including DEM.v1.00035533, which are the candidates for BBWV2 resistance. These findings provide promising genetic resources to help breed BBWV2-resistant pepper.

### Supplementary Information

Below is the link to the electronic supplementary material.

Supplementary Fig. 1 Box plots of ELISA results of resistant genetic resource accessions (GRAs). Resistant GRAs identified based on BBWV2 PAP1 inoculation were validated by ELISA. C. annuum ECW30R was used as susceptible control. All 30 GRAs were confirmed to be resistant. The box and lines indicate the median, first quartile, and third quartile values, while the x mark shows the mean value. One way ANOVA analysis followed by post hoc Tukey Kramer’s test was applied for statistical analysis, comparing every accession to ECW30R. All comparisons resulted in an adjusted p value < 0.001 (JPG 117 KB)

Supplementary Fig. 2 Aligned transcripts of the R pool and S pool in the 5′ UTR of DEM.v1.00035533 visualized by Integrative Genomics Viewer. Black boxes indicate exons and gray boxes indicate UTRs. Red line indicates the position of the intragenic SNP discovered by BSR seq. Each pool's read range is displayed from 0 to 1,394 reads, while zoomed in boxes range from 0 to 400 reads. The depth of reads drops significantly a t the location of the SNP from 88 to 24 in R pool transcripts. The read depth of S pool transcripts was 357 in the same location (JPG 79 KB)

Supplementary Fig. 3 Multiple sequence alignment of the 5′ UTR of DEM.v1.00035533. Sequence alignment between Dempsey, ECW, C V3, Maor, Thaihot, Micropep, Perennial and SNU C. Sequence alignment of the 5′ UTR of DEM.v1.00035533 between SNU C and ECW showed 86.55% identity. Most of the sequences are conserved in the seven genomes except SNU C. The red box indicates the SNU C speci fic variant found by BSR seq analysis. Other variants were not identified by BSR seq analysis due to the lack of transcription (JPG 499 KB)

Supplementary Fig. 4 Protein functional enrichment analysis of upregulated DEGs in the S pool. Annotation of upregulated DEGs r evealed major responses to pathogen invasion, such as response to oxygen containing compound, plant hormone responses (jasmonic acid), and defense response. The enriched proteins were ordered by the fold changes in the DEG set compared to the reference set (JPG 84 KB)

Supplementary file5 (XLSX 115 KB)

## Data Availability

The datasets generated and analyzed during the current study are available from the corresponding author on reasonable request.
