# Supplementary figures and images for: Molecular mapping of the broad bean wilt virus 2 resistance locus bwvr in Capsicum annuum using BSR-seq
Source: Theor Appl Genet. 2024 Apr 8;137(5):97. doi: 10.1007/s00122-024-04603-2 (PMC11001752; doi:10.1007/s00122-024-04603-2)

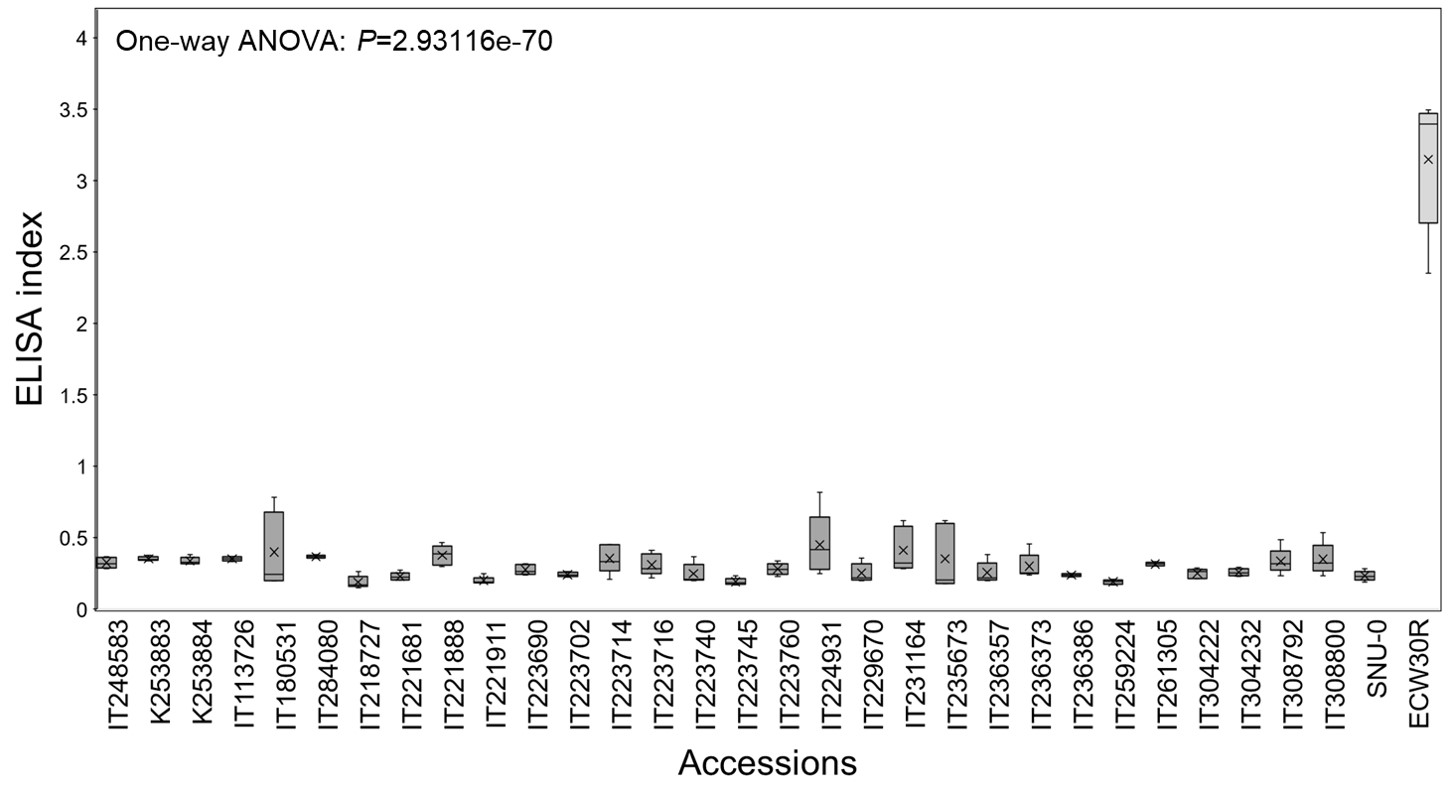

Supplement: Supplementary file 1 — Box plots of ELISA results of resistant genetic resource accessions (GRAs). Resistant GRAs identified based on BBWV2 PAP1 inoculation were validated by ELISA. C. annuum ECW30R was used as susceptible control. All 30 GRAs were confirmed to be resistant. The box and lines indicate the median, first quartile, and third quartile values, while the x mark shows the mean value. One way ANOVA analysis followed by post hoc Tukey Kramer’s test was applied for statistical analysis, comparing every accession to ECW30R. All comparisons resulted in an adjusted p value < 0.001 (JPG 117 KB) [file 122_2024_4603_MOESM1_ESM.jpg]

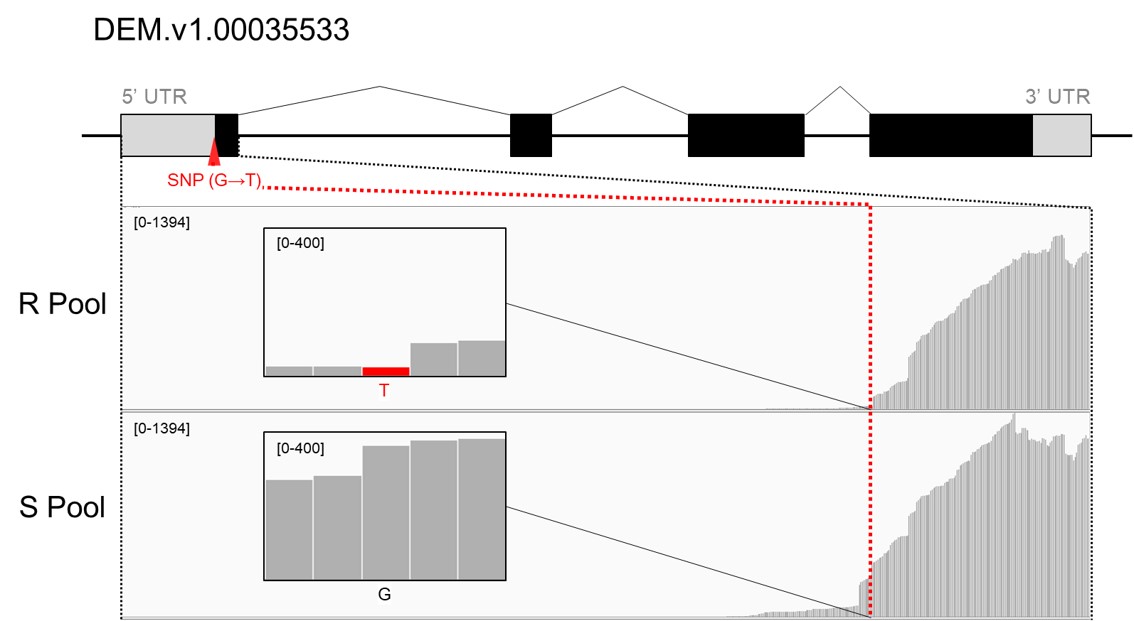

Supplement: Supplementary file 2 — Aligned transcripts of the R pool and S pool in the 5′ UTR of DEM.v1.00035533 visualized by Integrative Genomics Viewer. Black boxes indicate exons and gray boxes indicate UTRs. Red line indicates the position of the intragenic SNP discovered by BSR seq. Each pool's read range is displayed from 0 to 1,394 reads, while zoomed in boxes range from 0 to 400 reads. The depth of reads drops significantly a t the location of the SNP from 88 to 24 in R pool transcripts. The read depth of S pool transcripts was 357 in the same location (JPG 79 KB) [file 122_2024_4603_MOESM2_ESM.jpg]

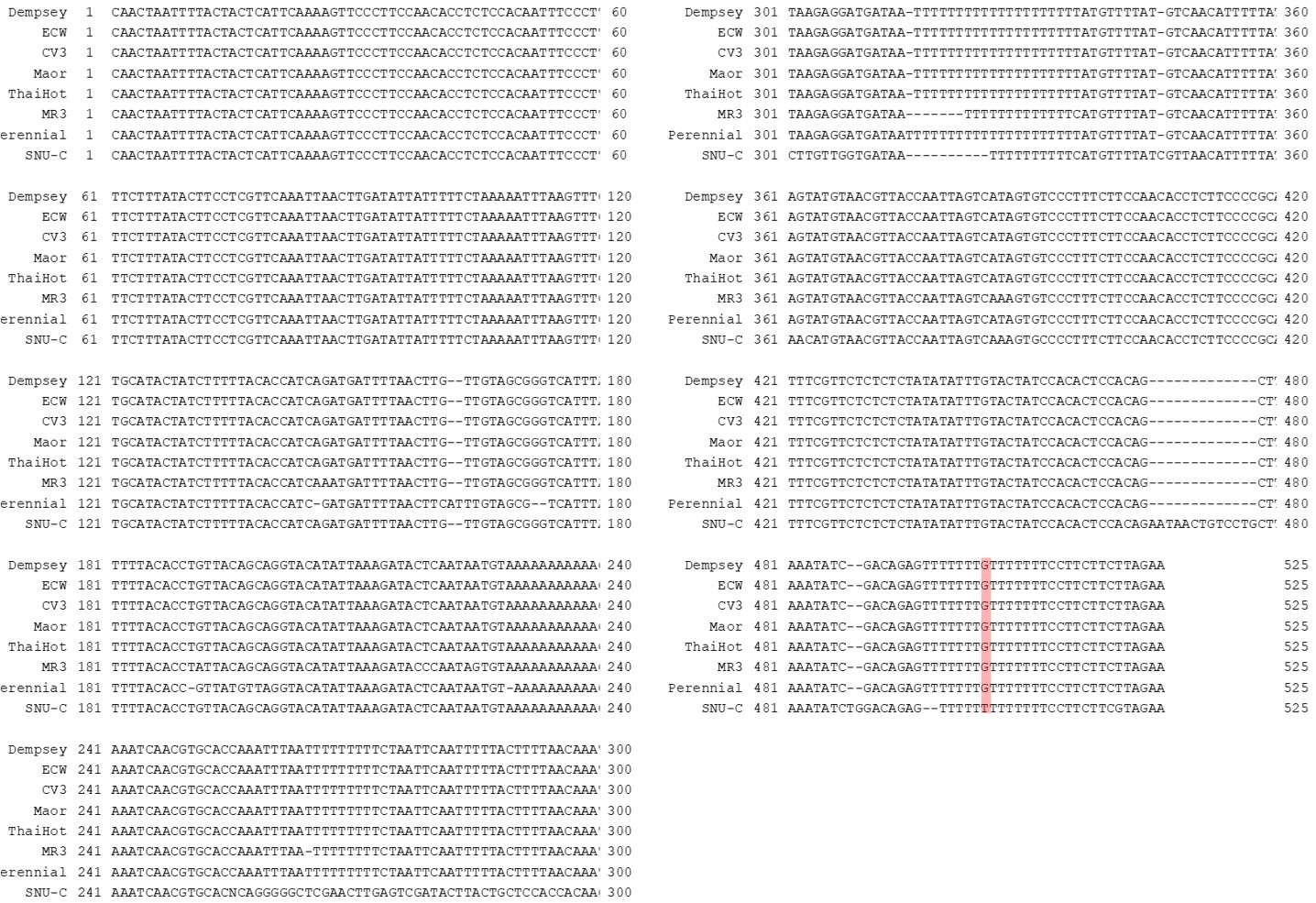

Supplement: Supplementary file 3 — Multiple sequence alignment of the 5′ UTR of DEM.v1.00035533. Sequence alignment between Dempsey, ECW, C V3, Maor, Thaihot, Micropep, Perennial and SNU C. Sequence alignment of the 5′ UTR of DEM.v1.00035533 between SNU C and ECW showed 86.55% identity. Most of the sequences are conserved in the seven genomes except SNU C. The red box indicates the SNU C speci fic variant found by BSR seq analysis. Other variants were not identified by BSR seq analysis due to the lack of transcription (JPG 499 KB) [file 122_2024_4603_MOESM3_ESM.jpg]

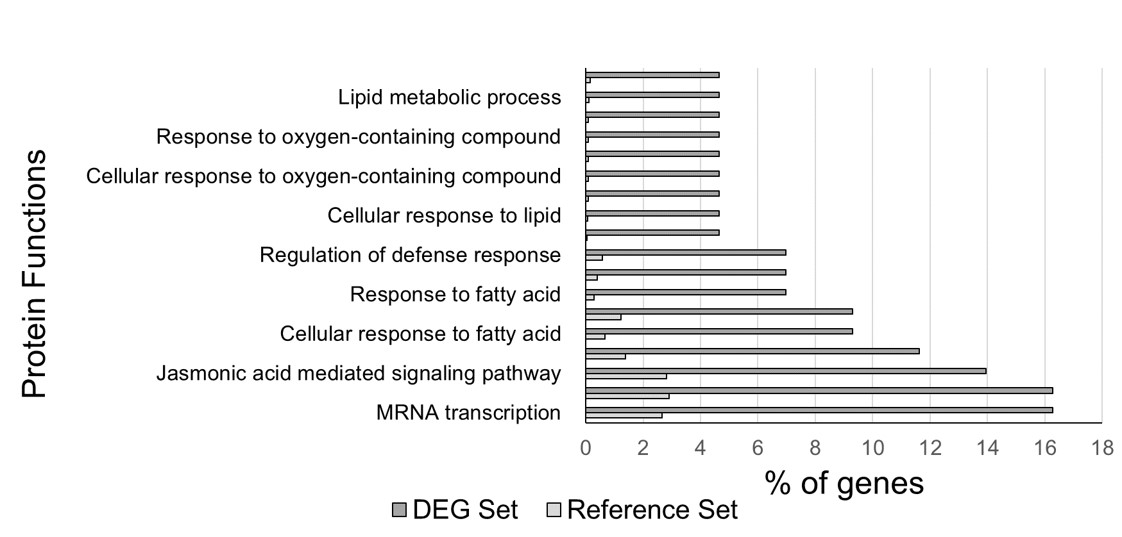

Supplement: Supplementary file 4 — Protein functional enrichment analysis of upregulated DEGs in the S pool. Annotation of upregulated DEGs r evealed major responses to pathogen invasion, such as response to oxygen containing compound, plant hormone responses (jasmonic acid), and defense response. The enriched proteins were ordered by the fold changes in the DEG set compared to the reference set (JPG 84 KB) [file 122_2024_4603_MOESM4_ESM.jpg]
